# The obesity puzzle: focus on bone turnover after bariatric surgery

**DOI:** 10.1590/2359-3997000000298

**Published:** 2017-07-01

**Authors:** Maria Edna de Melo

**Affiliations:** 1 Hospital das Clínicas Faculdade de Medicina Universidade de São Paulo São Paulo SP Brasil Grupo de Obesidade e Síndrome Metabólica, Hospital das Clínicas da Faculdade de Medicina da Universidade de São Paulo (HCFMUSP), São Paulo, SP, Brasil

This issue of the *Archives of Endocrinology and Metabolism* encompasses three studies related to obesity. The influence of genetics on the development of metabolic obesity complications, quality of life of obese patients and bone metabolism after bariatric surgery are discussed in order to increase the knowledge of these aspects in this complex disease.

The article “Prevalence of musculoskeletal symptoms in obese patients candidates for bariatric surgery and its impact on health related quality of life” by Calenzani and cols. emphasizes the relationship between obesity and musculoskeletal symptoms and their influence on physical and mental aspects linked to the quality of life. In addition to the classical metabolic disorders related to obesity that abbreviate patients’ lives, the physical limitation caused by arthritis and joint deformities causes impairment in mobility, increasing sedentary lifestyle, with consequent reduction in energy expenditure (1). Obese patients reported a very high frequency of pain in ankles/feet, knees and lumbar region. These symptoms impair deambulation and, thus obesity withdraws the right to come and go of these patients, determining furthermore impairment in their quality of life (1). Differently from Brilmann and cols. (2), who found a negative correlation between body mass index (BMI) and quality of life scores assessed through SF-36, Calenzani and cols. did not observed this association, that could be consequence of the relatively small and homogenously class 3 obesity sample of patients, as emphasized by the authors (1).

The relationship between two main metabolic disorders, diabetes mellitus type 2 (DM2) and systemic arterial hypertension (SAH), with 12 polymorphisms in the genes encoding ghrelin (GHRL, rs26802), uncoupling protein 2 (UCP2, rs659366), uncoupling protein 3 (UCP3, rs1800849), FTO (rs9939609), leptin (LEP, rs7799039), leptin receptor (LEPR, rs1137101), and serotonin receptor (5-HT2C, rs3813929) was investigated by Schnor and cols. and is reported in the paper “Association of 5-HT2C (rs3813929) and UCP3 (rs1800849) gene polymorphisms with type 2 diabetes in obese women candidates for bariatric surgery”. The study, which evaluated 351 obese women candidates for bariatric surgery, reports a 5-fold increased risk of having DM2 in T allele carriers of the rs3813929 and a 3-fold increased risk also for DM2 in CC genotype carriers of the rs1800849 (3). Analyzes of polymorphic variants can frequently lead to conflicting results, either because of the sample size or mainly because of the genetic background. The genetic influence on complex diseases is more associated with the interaction of several polymorphisms than with isolated polymorphisms. Thus, while one single published article reported a similar finding concerning the polymorphism in the UCP3 gene, studies about the polymorphism in the 5-HT2C gene present controversial results (3).

In the triad of articles on obesity is also included the study “Bone turnover after bariatric surgery” by Melo and cols., in which patients who had undergone bariatric surgery have an increase in bone turnover, characterized by elevated bone alkaline phosphatase and deoxypyridinoline (4). Interestingly, a high bone mineral density (BMD) was classically propagated as perhaps one of the few benefits of being overweight; however, more recently the mass of adipose tissue was negatively implicated in the bone microarchitecture (5).

The relationship between obesity and bone density and/or trabecular bone quality is influenced by body weight load, as well as cytokines and hormones such as insulin and leptin that are synthesized proportionally to the mass of white adipose tissue, with reducing levels after weight loss (4). In addition, patients with DM2 present a significant increase in cortical bone porosity in the radius, as well as a larger volume of the pores in the tibia, even with elevated BMD (6). Thus, the development of research in this area is of paramount importance, especially recently when a reduction in the BMI cutoff point is suggested for indication of bariatric surgery (7).

Maghrabi and cols. reported a reduction of more than 9% in femoral bone mineral density two years after bariatric surgery, regardless of the surgical procedure (Roux-en-Y gastric bypass or vertical gastrectomy). However, what is remarkable is that 6/37 (16.2%) patients in surgery group experienced spontaneous fractures, which occurred in only 1/17 (5.9%) in the control group (8). Weight loss, even with lifestyle changes, is associated with a reduction in BMD, as observed in patients participating in the LookAhead Trial (9). As a matter of fact, bariatric surgery determines a state of controlled malnutrition and thus, in addition to weight reduction, patients commonly present with vitamin D deficiency and difficulty in calcium absorption (4), which occurs preferentially in the duodenum, excluded from food transit in the gastric bypass operation.

An important point highlighted by Melo and cols. is the poor compliance to oral supplementation of vitamins and minerals, which strengthens the maintenance of nutritional deficiencies, such as hypovitaminosis D (4). Nevertheless, the most relevant finding in this study is the persistence of increased bone turnover even 7 years postoperatively despite the plateau in weight loss that tends to occur twelve months after the procedure. The influence of reduced levels of leptin after weight loss, implying high NTX (N-telopeptide of type 1 collagen) and low FGF23 (fibroblast growth factor 23) levels, could attenuate nutritional deficiencies, but the controversial leptin effect on BMD and bone microarchitecture calls for the development of further studies (4).

Not only weight loss is related to increased risk of bone fractures, but also some drugs used in the treatment of DM2 are associated with bone fracture, such as thiazolidinediones (OR 1.94 [95% CI 1.60, 2.35]) and SGLT2 inhibitors (1.32 [95% CI 1.00, 1.74]), particularly canagliflozin (9). While thiazolidinediones possibly inhibit osteoblasts differentiation and stimulate osteoclasts, increasing reabsorption (10), SGLT2 inhibitors may raise serum phosphate levels, implying in an elevation of FGF23 and, consequently, inhibition of the formation of calcitriol (9). In addition, in diabetic patients the occurrence of hypoglycemia and complications related to DM2, like microangiopathy, increase the risk of falls and consequently bone fractures (9).

Faced with the intricate complexity of obesity, each new published study represents a small piece in this vast puzzle.
